# Supplementary material for: Non‐enzymatic glycolysis and pentose phosphate pathway‐like reactions in a plausible Archean ocean
Source: Mol Syst Biol. 2014 Apr 25;10(4):725. doi: 10.1002/msb.20145228 (PMC4023395; doi:10.1002/msb.20145228)
Supplement: Supplementary file 3 — Supplementary Table S3 [file MSB-10-4-725-s07.pdf]

Supplementary Table S3: Ion source settings, Agilent 6460

Name	Value
**Scan Type**	MRM (SRM)
**Cell Acceleration voltage**	7 V
**Gas flow**	8 l/min
**Gas temperature**	300°C
**Sheath gas flow**	11 l/min
**Sheath gas temperature**	300°C
**Nebulizer**	50 psi (nitrogen)
**Negative Capillary voltage**	3000 V
**Nozzle voltage**	500 V
