# Supplementary material for: Non‐enzymatic glycolysis and pentose phosphate pathway‐like reactions in a plausible Archean ocean
Source: Mol Syst Biol. 2014 Apr 25;10(4):725. doi: 10.1002/msb.20145228 (PMC4023395; doi:10.1002/msb.20145228)
Supplement: Supplementary file 4 — Supplementary Table S4 [file MSB-10-4-725-s08.pdf]

Supplementary Table S4: SRM transitions and mass spectrometer parameters

Name	Sum formula	Exact Mass g/mol	Transition	Fragmentor	Collision energy	ESI Mode
**Glucose**	$C_6H_{12}O_6$	180.063388116	179.0 -> 89.0	70	1	Negative
**Pyr**	$C_3H_4O_3$	88.016043994	87.0 -> 43.0	55	3	Negative
**S7P**	$C_7H_{15}O_{10}P$	290.040283212	289.0 -> 97.0	100	12	Negative
**G6P**	$C_6H_{13}O_9P$	260.029718526	259.0 -> 97.0	100	12	Negative
**X5P/Ru5P**	$C_5H_9O_8P$	230.01915384	229.0 -> 97.0	85	12	Negative
**F6P**	$C_6H_{13}O_9P$	260.029718526	259.0 -> 97.0	100	12	Negative
**E4P**	$C_4H_9O_7P$	200.008589154	199.0 -> 97.0	70	6	Negative
**G3P**	$C_3H_7O_6P$	169.998024468	169.0 -> 97.0	70	5	Negative
**R5P**	$C_5H_9O_8P$	230.01915384	229.0 -> 97.0	85	12	Negative
**DHAP**	$C_3H_7O_6P$	169.998024468	169.0 -> 97.0	70	5	Negative
**6PG**	$C_6H_{13}O_{10}P$	276.024633148	275.0 -> 97.0	100	18	Negative
**2-PG/3-PG**	$C_3H_7O_7P$	185.99293909	185.0 -> 97.0	75	11	Negative
**PEP**	$C_3H_5O_6P$	167.982374404	167.0 -> 79.0	50	7	Negative
**F16BP**	$C_6H_{14}O_{12}P_2$	339.996048936	339.0 -> 97.0	175	16	Negative
